# Update on Vaccine-Derived Poliovirus Outbreaks — Worldwide, July 2019–February 2020

**DOI:** 10.15585/mmwr.mm6916a1

**Published:** 2020-04-24

**Authors:** Mary M. Alleman, Jaume Jorba, Sharon A. Greene, Ousmane M. Diop, Jane Iber, Graham Tallis, Ajay Goel, Eric Wiesen, Steven G.F. Wassilak, Cara C. Burns

**Affiliations:** ^1^Global Immunization Division, Center for Global Health, CDC; ^2^Division of Viral Diseases, National Center for Immunization and Respiratory Diseases, CDC; ^3^Epidemic Intelligence Service, CDC; ^4^Polio Eradication Department, World Health Organization, Geneva, Switzerland.

Circulating vaccine-derived polioviruses (cVDPVs) can emerge in areas with low poliovirus immunity and cause outbreaks* of paralytic polio (*1*–*5*). Among the three types of wild poliovirus, type 2 was declared eradicated in 2015 (*1*,*2*). The use of trivalent oral poliovirus vaccine (tOPV; types 1, 2, and 3 Sabin strains) ceased in April 2016 via a 1-month–long, global synchronized switch to bivalent OPV (bOPV; types 1 and 3 Sabin strains) in immunization activities (*1*–*4*). Monovalent type 2 OPV (mOPV2; type 2 Sabin strain) is available for cVDPV type 2 (cVDPV2) outbreak response immunization (*1*–*5*). The number and geographic breadth of post-switch cVDPV2 outbreaks have exceeded forecasts that trended toward zero outbreaks 4 years after the switch and assumed rapid and effective control of any that occurred (*4*). New cVDPV2 outbreaks have been seeded by mOPV2 use, by both suboptimal mOPV2 coverage within response zones and recently mOPV2-vaccinated children or contacts traveling outside of response zones, where children born after the global switch are fully susceptible to poliovirus type 2 transmission (*2*–*4*). In addition, new emergences can develop by inadvertent exposure to Sabin OPV2-containing vaccine (i.e., residual response mOPV2 or tOPV) (*4*). This report updates the January 2018–June 2019 report with information on global cVDPV outbreaks during July 2019–February 2020 (as of March 25, 2020)^†^ (*2*). Among 33 cVDPV outbreaks reported during July 2019–February 2020, 31 (94%) were cVDPV2; 18 (58%) of these followed new emergences. In mid-2020, the Global Polio Eradication Initiative (GPEI) plans to introduce a genetically stabilized, novel OPV type 2 (nOPV2) that has a lower risk for generating VDPV2 than does Sabin mOPV2; if nOPV2 is successful in limiting new VDPV2 emergences, GPEI foresees the replacement of Sabin mOPV2 with nOPV2 for cVDPV2 outbreak responses during 2021 (*2*,*4*,*6*).

## Detection of cVDPV Type 1

**FIGURE 1:**
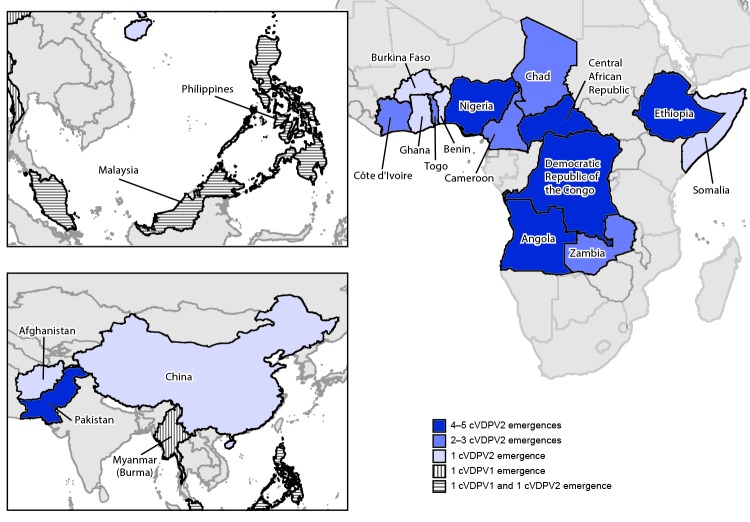
Ongoing circulating vaccine-derived poliovirus (cVDPV) outbreaks — worldwide, July 2019–February 2020* **Abbreviations: **cVDPV1 = cVDPV type 1; cVDPV2 = cVDPV type 2. * Data as of March 24–27, 2020.

No poliovirus genetically linked to the Papua New Guinea cVDPV type 1 (cVDPV1) emergence (PNG-MOR-1^§^) was detected after November 2018 (*1*,*2*). In Indonesia, the most recent cVDPV1 outbreak isolate was from February 2019 (IDN-PAP-1), and in Myanmar (Burma), the most recent were from August 2019 (MMR-KAY-1) (*2*) (Table) (Figure 1). During the reporting period, a new cVDPV1 emergence (PHL-NCR-2) was first detected in environmental surveillance (sewage) samples collected in July 2019 in the National Capital Region of the Philippines. Genetically linked virus was isolated from sewage samples collected in Sabah State, Malaysia, in June and November 2019; however, delays in sample processing resulted in findings not being released until December 2019. The most recent isolate linked to PHL-NCR-2 was detected in a specimen from a patient from Malaysia with acute flaccid paralysis (AFP) onset in January 2020.

**TABLE:** Circulating vaccine-derived polioviruses (cVDPVs) detected, by serotype, source and other selected characteristics — worldwide, July 2019–February 2020

Country	Emergence designation*	Years detected^†^	Serotype	No. of isolates^§^ July 2019–February 2020			Capsid protein VP1 divergence from Sabin OPV strain**(%)	Date of latest outbreak case, healthy child specimen, or environmental sample^††^
				From AFP cases	From other human sources (non-AFP)^¶^	From environmental surveillance		
Afghanistan	PAK-GB-1	2020	2	0	0	10	1.1–2.0	Feb 5, 2020
Angola	ANG-HUI-1	2019–2020	2	76	2	13	0.7–1.8	Feb 9, 2020
Angola	ANG-LNO-2	2019	2	14	1	0	1.1–2.2	Dec 25, 2019
Angola	ANG-MOX-1	2019	2	12	2	0	0.8–1.6	Dec 18, 2019
Angola	ANG-LUA-1	2019	2	34	3	14	0.7–1.5	Dec 27, 2019
Benin	NIE-JIS-1	2019–2020	2	8	0	0	3.3	Jan 16, 2020
Burkina Faso	NIE-JIS-1	2019–2020	2	1	3	0	3.7	Jan 11, 2020
Cameroon	CHA-NDJ-1	2019	2	0	0	2	1.1	Dec 16, 2019
Cameroon	NIE-JIS-1	2019	2	0	0	1	3.3	Dec 2, 2019
Cameroon	CAR-BNG-1	2020	2	1	0	0	2.2	Jan 30, 2020
CAR	CAR-BAM-1	2019	2	3	2	6	0.8–2.1	Nov 20, 2019
CAR	CAR-BER-1	2019	2	3	3	1	0.8–1.2	Dec 8, 2019
CAR	CAR-BIM-2	2019	2	0	0	3	1.3–2.2	Sep 11, 2019
CAR	CAR-BIM-3	2019	2	2	7	0	0.8–1.6	Aug 23, 2019
CAR	CAR-BNG-1	2019–2020	2	9	3	10	0.7–1.9	Feb 5, 2020
Chad	NIE-JIS-1	2019–2020	2	5	7	2	2.6–4.5	Feb 5, 2020
Chad	CHA-NDJ-1	2019–2020	2	8	3	10	0.7–2.5	Feb 5, 2020
China	CHN-XIN-1	2018–2019	2	0	1	0	3.0	Aug 18, 2019
Côte d’Ivoire	NIE-JIS-1	2019–2020	2	0	0	31	2.8–4.0	Feb 11, 2020
Côte d’Ivoire	TOG-SAV-1	2020	2	1	0	0	2.0	Feb 10, 2020
DRC	DRC-HLO-2	2019	2	13	5	0	1.0–1.7	Dec 13, 2019
DRC	DRC-KAS-3	2019–2020	2	18	6	0	1.3–2.2	Feb 8, 2020
DRC	DRC-SAN-1	2019	2	26	1	0	0.7–1.8	Nov 30, 2019
DRC	ANG-LUA-1	2019–2020	2	12	3	0	0.7–1.3	Jan 22, 2020
Ethiopia	SOM-BAN-1	2019	2	3	0	0	5.4–5.6	Aug 13, 2019
Ethiopia	ETH-ORO-1	2019–2020	2	11	3	1	1.1–2.6	Feb 12, 2020
Ethiopia	ETH-ORO-2	2019–2020	2	3	0	0	1.2–1.5	Jan 26, 2020
Ethiopia	ETH-ORO-3	2019–2020	2	1	1	0	2.0–2.2	Feb 21, 2020
Ethiopia	ETH-SOM-1	2019	2	0	1	2	1.5	Dec 30, 2019
Ghana	NIE-JIS-1	2019–2020	2	24	29	50	1.8–4.0	Feb 15, 2020
Malaysia	PHL-NCR-1	2019	2	0	0	2	6.8–7.1	Nov 19, 2019
Malaysia	PHL-NCR-2	2019–2020	1	3	0	8	3.6–3.9	Jan 24, 2020
Myanmar^§§^	MMR-KAY-1	2019	1	2	5	0	3.4–3.6	Aug 21, 2019
Nigeria	NIE-JIS-1	2018–2019	2	1	2	2	2.4–2.5	Oct 9, 2019
Nigeria	NIE-KGS-1	2019–2020	2	2	1	5	0.9–1.5	Jan 26, 2020
Nigeria	NIE-KGS-2	2019	2	1	3	0	0.7–0.8	Aug 8, 2019
Nigeria	NIE-SOS-6	2019	2	0	0	1	1.1	Sep 11, 2019
Pakistan	PAK-GB-1	2019–2020	2	41	18	65	0.7–2.0	Feb 10, 2020
Pakistan	PAK-GB-2	2019	2	0	2	1	0.7–1.3	Aug 28, 2019
Pakistan	PAK-GB-3	2019	2	1	1	0	0.9–1.0	Aug 22, 2019
Pakistan	PAK-KOH-1	2019	2	1	1	2	0.7–1.3	Nov 12, 2019
Pakistan	PAK-TOR-1	2019–2020	2	2	4	4	0.7–1.5	Jan 3, 2020
Philippines	PHL-NCR-1	2019–2020	2	14	6	30	6.8–7.8	Jan 24, 2020
Philippines	PHL-NCR-2	2019	1	1	1	22	3.3–4.4	Nov 28, 2019
Somalia	SOM-BAN-1	2017–2020	2	0	0	10	5.7–6.4	Feb 4, 2020
Togo	NIE-JIS-1	2019–2020	2	11	1	0	2.7–4.1	Jan 23, 2020
Togo	TOG-SAV-1	2019–2020	2	3	2	0	1.4–1.9	Feb 1, 2020
Zambia	ZAM-LUA-1	2019	2	1	2	0	1.0–1.1	Sep 25, 2019
Zambia	ANG-MOX-1	2019	2	1	0	0	1.1	Nov 25, 2019
**Total cVDPV**	**—^¶¶^**	**—^¶¶^**	**—^¶¶^**	**373**	**135**	**308**	**—^¶¶^**	**—^¶¶^**

**Abbreviations:** AFP = acute flaccid paralysis; CAR = Central African Republic; DRC = Democratic Republic of the Congo; OPV = oral poliovirus vaccine.

* Outbreaks list total cases clearly associated with cVDPVs; emergences indicate independent cVDPV outbreaks and designate the location of the emergence and the number of emergences in a geographic region.

^†^ Total years detected.

^§^ Total VDPV-positive specimens obtained from AFP patients and total VDPV-positive environmental (sewage) samples as of March 25 2020, for all emergences except the following: 1) ETH-ORO-1, ETH-ORO-2, ETH-ORO-3, ETH-SOM-1, and SOM-BAN-1 (as of March 24, 2020) and 2) CHA-NDJ-1, NIE-JIS-1, NIE-KGS-1, NIE-KGS-2, NIE-SOS-6, and TOG-SAV-1 (as of March 27, 2020).

^¶^ Contacts and healthy child sampling as of March 25, 2020, for all emergences except for the following: 1) ETH-ORO-1, ETH-ORO-3, and ETH-SOM-1 (as of March 24, 2020) and 2) CHA-NDJ-1, NIE-JIS-1, NIE-KGS-1, NIE-KGS-2, and TOG-SAV-1 (as of March 27, 2020).

** Percentage of divergence is estimated from the number of nucleotide differences in the VP1 region from the corresponding parental OPV strain.

^††^ For AFP cases, dates refer to date of paralysis onset; for contacts, healthy children, and environmental (sewage) samples, dates refer to date of collection.

^§§ ^U.S. State Department country name is Burma.

^¶¶^ Not cumulative data.

## Detection of cVDPV2

**FIGURE 2:**
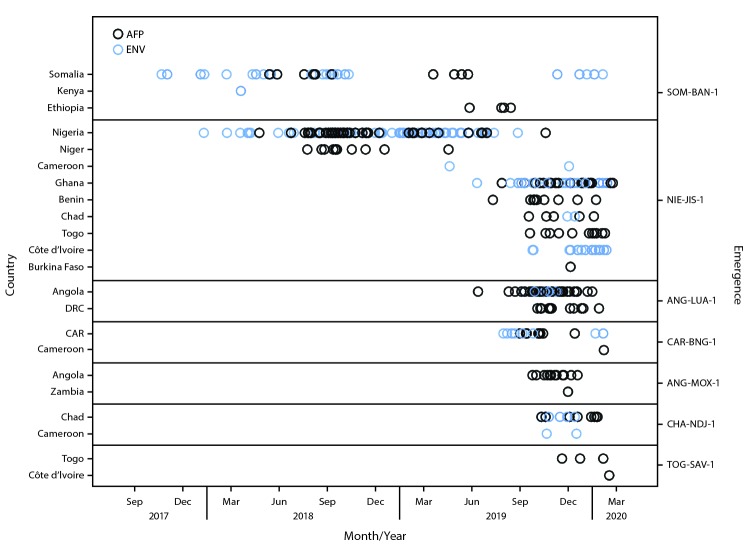
Acute flaccid paralysis (AFP) cases and environmental samples positive for circulating vaccine-derived poliovirus type 2 associated with outbreaks ongoing during July 2019–February 2020 that involved international spread since emergence, by outbreak and country — Africa, October 2017–February 2020*^,†^ **Abbreviation:** CAR = Central African Republic; DRC = Democratic Republic of the Congo; ENV = environmental surveillance. * Dates (month/year) refer to the date of specimen collection. For samples collected on the same dates, symbols will overlap; thus, not all isolates are visible. ^†^ Data as of March 25, 2020, for all emergences except the following: 1) SOM-BAN-1 (as of March 24, 2020) and 2) CHA-NDJ-1, NIE-JIS-1, and TOG-SAV-1 (as of March 27, 2020).

During July 2019–February 2020, among 31 active cVDPV2 outbreaks, 18 (58%) followed new emergences; one outbreak in Malaysia and the Philippines (PHL-NCR-1) was detected during the reporting period, although genetic sequencing analyses indicate that the emergence occurred years earlier and genetically linked viruses circulated undetected by surveillance (Table) (Figure 1) (*1*,*2*). Twenty-four (77%) of the 31 active outbreaks affected African countries; seven of these (29%) resulted in international spread (Figure 2).

**Western Africa.** The previously described cVDPV2 emergence in Nigeria (NIE-JIS-1) continued to circulate during the reporting period (*1*,*2*); the most recent NIE-JIS-1 isolations in Niger and Nigeria were detected among specimens from AFP patients in April and October 2019, respectively. Detection of genetically linked virus from AFP patients’ specimens and through environmental surveillance occurred in Benin, Burkina Faso, Cameroon, Chad, Côte d’Ivoire, Ghana, and Togo during the reporting period. Since its first detection in Nigeria in January 2018, NIE-JIS-1 emergence has resulted in 101 cases in seven countries (*1*,*2*). Ongoing transmission of previously reported cVDPV2 emergences (NIE-KGS-1 and NIE-KGS-2) and of a new cVDPV2 emergence (NIE-SOS-6) was detected in Nigeria (*2*). No polioviruses genetically linked to other previously described emergences (NIE-SOS-3, NIE-SOS-4, and NIE-SOS-5) (*1*,*2*) were detected during the reporting period. A new emergence (TOG-SAV-1) in Togo was first detected in November 2019, and a genetically linked virus was isolated from a specimen obtained from an AFP patient in Côte d’Ivoire in February 2020.

**Central Africa.** Five Central African countries were affected by cVDPV2 outbreaks during July 2019–February 2020. Each country had a minimum of two cVDPV2 emergences circulating during the reporting period, with the Central African Republic (CAR) having five.

In Angola, no poliovirus genetically linked to the previously described cVDPV2 emergence (ANG-LNO-1) was detected after May 2019 (*2*). However, polioviruses genetically linked to previously described emergences (ANG-HUI-1 and ANG-LNO-2) continued to circulate during the reporting period within Angola, resulting in 78 cases (ANG-HUI-1) and 15 cases (ANG-LNO-2) since first detection (*2*). In addition, two new emergences were detected in June (ANG-LUA-1) and September (ANG-MOX-1) 2019, resulting in a total of 46 cVDPV2 cases in Angola; the two emergences also circulated in the Democratic Republic of the Congo (DRC; ANG-LUA-1) and Zambia (ANG-MOX-1). The detection of concurrent and independent cVDPV2 emergences in Angola might be associated with mOPV2 response–related supplementary immunization activities (SIAs; vaccination campaigns) in neighboring DRC or related to other Sabin OPV2 inadvertent exposure in Angola; investigation is ongoing.

In CAR, the previously described CAR-BAM-1 and CAR-BIM-2 emergences continued to circulate during the reporting period, resulting in three cases and six detections of CAR-BAM-1 and three detections of CAR-BIM-2 through environmental surveillance (*2*). No polioviruses genetically linked to the previously described CAR-BAM-2 or CAR-BIM-1 emergences were detected after June 2019 (*2*). Three new emergences (CAR-BER-1, CAR-BIM-3, and CAR-BNG-1) were detected during the reporting period and resulted in a total of 14 cases in CAR. Virus genetically linked to CAR-BNG-1 was isolated from a specimen obtained from an AFP patient in Cameroon with paralysis onset in January 2020.

In Chad, circulation of a new emergence (CHA-NDJ-1) was first detected in October 2019. Genetically linked viruses were continually detected in specimens from AFP patients in Chad into 2020 and from environmental surveillance in Cameroon and Chad through the end of 2019.

In DRC, the previously described emergences, DRC-HLO-2, DRC-KAS-3, and DRC-SAN-1, continued to circulate, resulting in 20, 21, and 32 cases, respectively, since detection (*2*). During the reporting period, cVDPV2 genetically linked to the Angola ANG-LUA-1 emergence was detected in specimens obtained from 12 AFP patients in DRC. No evidence of continued circulation of the other previously described emergences (DRC-HKA-1, DRC-HLO-1, DRC-KAS-1, DRC-KAS-2, DRC-MAN-1, DRC-MON-1, and DRC-TPA-1) was found (*1*,*2*).

**Southern Africa.** In Zambia, the ZAM-LUA-1 emergence was detected in specimens obtained from an AFP patient and two contacts during July–September 2019. In addition, cVDPV2 linked to the ANG-MOX-1 emergence was detected in a specimen obtained from an AFP patient with paralysis onset in November 2019. In Mozambique, no transmission related to the previously described MOZ-ZAM-2 emergence has been detected since December 2018 (*2*).

**Horn of Africa.** During July 2019–February 2020, cVDPV2 genetically related to the previously described SOM-BAN-1 emergence, which was first detected in October 2017 in Banadir Province, Somalia (*1*–*3*), continued to circulate. During this reporting period, genetically linked virus was detected from specimens from three AFP patients in Ethiopia and in 10 sewage samples from Banadir. In Ethiopia, four new cVDPV2 emergences (ETH-ORO-1, ETH-ORO-2, ETH-ORO-3, and ETH-SOM-1) were detected during this period among specimens from 15 AFP patients and through environmental surveillance in Addis Ababa and the Somali region.

**Pakistan and Afghanistan**. The PAK-GB-1 emergence was the first of five total cVDPV2 emergences (PAK-GB-1, PAK-GB-2, PAK-GB-3, PAK-KOH-1, and PAK-TOR-1) detected in Pakistan during the reporting period. PAK-GB-1 has resulted in 41 AFP cases in Pakistan and has been isolated through environmental surveillance in Pakistan and Afghanistan as recently as February 2020. The last detections of the PAK-GB-2 and PAK-GB-3 cVDPV2s were in August 2019. PAK-KOH-1 and PAK-TOR-1 emergences were detected from specimens obtained from AFP patients and through environmental surveillance during September 2019–January 2020. Current genetic evidence indicates that the 2016 mOPV2 outbreak response SIAs in Pakistan did not initiate these cVDPV2 outbreaks. Possible origins include international importations from areas using mOPV2 or inadvertent use of residual tOPV or mOPV2 (*4*).

**China**. The CHN-XIN-1 emergence was first isolated through environmental surveillance in Xinjiang province in April 2018; genetically linked virus was last detected in Sichuan province in August 2019 from the stool specimen of a community contact of an AFP patient who had paralysis onset in April 2019 (*2*).

**Malaysia and the Philippines.** During the reporting period, the PHL-NCR-1 emergence was identified from a specimen obtained from an AFP patient with paralysis onset in June 2019 in Mindanao Province, the Philippines. Subsequently, genetically linked virus was detected among specimens from 13 additional AFP patients in the Philippines and through environmental surveillance in both Malaysia and the Philippines during July 2019–February 2020.

## Outbreak Control

As of the end of February 2020, no transmission was detected for ≥13 months for previously reported outbreaks related to one cVDPV1 emergence in Papua New Guinea (PNG-MOR-1), one cVDPV3 emergence in Somalia (SOM-BAN-2), and six cVDPV2 emergences in DRC (DRC-HLO-1, DRC-MAN-1, DRC-MON-1, and DRC-HKA-1), Mozambique (MOZ-ZAM-2), and Syria (designation not assigned), indicating probable outbreak cessation (*1*–*3*,*5*,*7*). Emergences of cVDPV in Angola (ANG-LNO-1); CAR (CAR-BAM-2 and CAR-BIM-1); DRC (DRC-KAS-1, DRC-KAS-2, and DRC-TPA-1); Indonesia (IDN-PAP-1); and Nigeria (NIE-SOS-3, NIE-SOS-4, and NIE-SOS-5) have had no genetically linked isolations for 7–12 months, indicating possible outbreak cessation (*1*,*2*,*5*,*7*).

## Discussion

After outbreak detection, prompt and effective mOPV2 vaccination of children will interrupt cVDPV2 transmission and limit emergence of new VDPV2 strains in outbreak response zones. Although many previously identified cVDPV2 outbreaks have been interrupted or controlled as forecasted (*1*–*4*), GPEI has been challenged by the increased number of outbreaks from newly seeded VDPV2 emergences during January 2018–February 2020, following mOPV2 SIAs that did not reach sufficient coverage; in addition, there are protracted cVDPV2 outbreaks from prior emergence that have not been successfully controlled for the same reason (*1*–*4*). In areas where no mOPV2 has yet been used, approximately four birth cohorts that are fully susceptible to mucosal poliovirus type 2 infection have accumulated since the April 2016 tOPV-to-bOPV switch (*1*,*2*,*4*).

The utility of environmental surveillance to complement AFP surveillance has been demonstrated by detections of continued circulation after a long absence in detection of confirmed AFP cases (e.g., SOM-BAN-1 in Somalia) and of circulation before detection of confirmed AFP cases (e.g., NIE-JIS-1 in Ghana); some outbreak transmission has been detected only through environmental surveillance (e.g., NIE-SOS-6 in Nigeria) (*8*).

To address these challenges, GPEI adopted the 2020–2021 Strategy for the Response to Type 2 Circulating Vaccine-Derived Poliovirus as an addendum to the Polio Endgame Strategy 2019–2023 (*6*). The response strategy aims to improve the quality of mOPV2 SIAs through enhanced technical support, enactment of full international health emergency procedures, and enhanced population protection from paralysis through periodic intensification of routine immunization with bOPV and injectable inactivated poliovirus vaccine. After accelerated development and clinical testing of nOPV2 (*9*), which has a substantially lower risk for reversion to neurovirulence (*2*,*9*), this vaccine is expected to be available in mid-2020 for initial outbreak responses under emergency use listing requirements (*10*). If wider outbreak response use is allowed and ample supplies are available by the end of 2020, nOPV2 will replace Sabin mOPV2 in outbreak response to prevent new VDPV2 emergences (*6*). This time line and the course of ongoing and newly emergent cVDPV outbreaks could be negatively affected during the coronavirus disease 2019 (COVID-19) pandemic because of changes in priorities for use of health care resources and decreased immunization activities.^¶^ Cessation of all OPV use after certification of polio eradication will eliminate the risk of VDPV emergence (*2*,*4*).

**Summary**

**What is already known about this topic?**

Circulating vaccine-derived polioviruses (cVDPVs) can emerge in settings with low poliovirus immunity and can cause paralysis.

**What is added by this report?**

Thirty-one ongoing and new cVDPV type 2 (cVDPV2) outbreaks were documented during July 2019–February 2020; nine outbreaks spread internationally. New cVDPV2 outbreaks were often linked to poor coverage with monovalent Sabin oral poliovirus vaccine (OPV) type 2 during outbreak response campaigns.

**What are the implications for public health practice?**

The Global Polio Eradication Initiative plans to introduce a genetically stabilized, novel OPV type 2 for outbreak response in mid-2020 and expand use in 2021. Cessation of all OPV use after certification of polio eradication will eliminate the risk of VDPV emergence.

## Footnotes

* In this report, a cVDPV outbreak is defined as two or more independent isolations (through acute flaccid paralysis [AFP] or environmental surveillance or from a healthy community member following a confirmed AFP case) of genetically linked VDPVs. The number of outbreaks is equivalent to the number of cVDPV emergences. In summaries, a given cVDPV emergence/outbreak is counted once regardless of the number of countries affected following transmission beyond national borders.

† Data as of March 25, 2020, for all emergences except ETH-ORO-1, ETH-ORO-2, ETH-ORO-3, ETH-SOM-1, and SOM-BAN-1, (as of March 24, 2020) and CHA-NDJ-1, NIE-JIS-1, NIE-KGS-1, NIE-KGS-2, NIE-SOS-6, and TOG-SAV-1 (as of March 27, 2020).

§ Names designate the country and geographic region of the emergence and the number of emergences in each geographic region.

¶ GPEI has offered its global technical and material assets to support the COVID-19 pandemic response and has recommended that preventive and response polio SIAs be suspended until June 1, 2020, or later. AFP and environmental surveillance activities should continue to the extent possible and according to countries’ COVID-19 contexts, as well as preparations for the use of nOPV2. http://polioeradication.org/news-post/global-polio-eradication-and-covid-19/.
